# Beet Chlorosis Virus Infection Mitigates Aphid‐Induced Plant Defences and Improves Plant Acceptability to Aphid Vectors

**DOI:** 10.1111/mec.70092

**Published:** 2025-08-29

**Authors:** Thomas Armand, Sylvaine Boissinot, Alessandra Maia‐Grondard, Philippe Hugueney, Véronique Brault, Quentin Chesnais

**Affiliations:** ^1^ INRAE Université de Strasbourg, SVQV Colmar France

**Keywords:** host parasite interactions, insects, invertebrates, species interactions

## Abstract

Plant viruses often alter host traits in ways that affect interactions with herbivores, potentially facilitating their own acquisition and transmission by insect vectors. However, little is known about the molecular mechanisms underlying this phenomenon. This is particularly true for agronomically important pathosystems, such as the viruses responsible for sugar beet yellowing. Among them is the beet chlorosis virus (BChV), whose effects on aphid vector behaviour and plant defence mechanisms have not been fully characterised. In this study, we demonstrate that BChV infection suppresses sugar beet defences induced by aphid pre‐infestation, enhancing plant acceptability for aphids. Specifically, gene expression analyses revealed a downregulation of the aphid‐induced ethylene pathway in infected plants, along with alterations in the salicylic acid pathway that may benefit aphids. Metabolic profiling highlighted reduced levels of phenolic acids, including cinnamic and coumaric acids, in virus‐infected plants which likely contribute to increased plant acceptability by aphids. By integrating gene expression, metabolic profiling, and behavioural assays, our findings illustrate how BChV manipulates host–plant defences to potentially increase its transmission by aphids, underscoring the broad ecological and evolutionary significance of virus‐mediated plant–vector interactions.

## 1 Introduction

Plants are frequently subjected to simultaneous biotic stresses, creating synergistic or antagonistic interactions that challenge plant defences (Basu et al. 2021; Fernández de Bobadilla et al. 2022). For example, co‐occurrence of both herbivorous insects and pathogens on the same plant is, at least temporarily, required for successful acquisition and inoculation of insect‐borne viruses.

Plant defences are regulated by a few key phytohormones, including salicylic acid (SA), jasmonic acid (JA), and ethylene (ET), which mediate responses to different attackers. JA is primarily involved in plant defences against necrotrophic pathogens and chewing insects, while SA mediates responses to biotrophic pathogens and phloem‐feeding insects (Lazebnik et al. 2014). Ethylene plays a complex role, indirectly modulating SA‐ and JA‐dependent pathways (Broekgaarden et al. 2015), although recent studies highlight its direct involvement in plant defences against aphids (Louis et al. 2015) and viruses (Zhao et al. 2022).

When plants face simultaneous biotic stresses, they must prioritise their defence responses, often leading to ‘phytohormone cross‐talk’. This cross‐talk can result in resource reallocation and/or the mobilisation of defences that may affect associated attackers (Aerts et al. 2021). Beyond phytohormonal adjustments, biotic aggressors can alter the nutritional quality of plants (e.g., amino acid accumulation in the plant (Mauck et al. 2014; Patton et al. 2020)) with a downstream effect on pathogens and herbivorous insect performances (e.g., Ángeles‐López et al. 2016; Basu et al. 2021).

A growing body of evidence indicates that insect vector‐borne viruses can modify host plant phenotypes (primary and secondary metabolites, size, colour, volatile emissions and other traits) in ways that enhance interactions between the host plant and the insect vector, thereby facilitating viral transmission (Mauck et al. 2012). This phenomenon, known as ‘virus manipulation’, reflects the complex ecological interactions shaped by these pathogens. More specifically, virus indirect (plant‐mediated) effects on vector behavioural and physiological responses tend to differ depending on the pathogen localisation in the plant (phloem restricted vs. non‐phloem restricted viruses) and in the insect (stylet‐borne vs. circulative viruses) which determines the transmission mechanism (Eigenbrode et al. 2018; Mauck et al. 2018). For example, aphids spent a longer time ingesting phloem sap on cucumber infected by the phloem‐restricted and circulative cucurbit aphid‐borne yellows virus (CABYV, *Polerovirus*) (Carmo‐Sousa et al. 2016), but a shorter time on cucumber infected by the non‐phloem restricted and stylet‐borne cucumber mosaic virus (CMV, *Cucumovirus*) (Carmo‐Sousa et al. 2014). Direct effects on the vector behaviour following virus acquisition also tended to enhance virus transmission probability (Chesnais et al. 2020; Mauck et al. 2019; Verdier et al. 2023). These virus‐induced indirect or direct modifications on the vector behaviour may ultimately have epidemiological impacts on the dispersion of the virus‐induced diseases (Cunniffe et al. 2021).

Although virus‐induced changes in host plant phenotypes and vector behaviour have been studied for key viral families, relatively few studies have focused on the molecular and genetic mechanisms underlying the tripartite interactions between plants, viruses, and vectors. On the virus side, certain silencing suppressors have been shown to reduce plant defence pathways, thereby influencing vector preference or fitness (Mauck et al. 2019; Ziegler‐Graff 2020). Other viral proteins or components act as drivers of plant modifications that alter vector behaviour and performance. For example, three non‐structural proteins of the potato leafroll virus (PLRV, *Polerovirus*) have been shown to impact hormonal pathways at gene transcript and metabolic levels, significantly impacting aphid preferences and fecundity (Patton et al. 2020). Recently, large‐scale transcriptomic analyses have further identified pathways, such as RNA gene silencing and phytohormone synthesis, as potential contributors to virus‐mediated manipulation of plants infected with viruses and pre‐infested by aphids (Chesnais et al. 2022; Nachappa et al. 2020). Notably, in plants infected with turnip yellows virus (TuYV, *Polerovirus*), many aphid‐induced gene deregulations were alleviated, correlating with enhanced virus transmission (Krieger et al. 2023). These recent findings underscore the complexity of the tripartite interactions and the need to integrate molecular, genetic, metabolomic, and ecological approaches to unravel these mechanisms.

The phloem‐limited poleroviruses (family *Solemoviridae*) and their aphid vectors may serve as valuable models for exploring plant–virus–vector intricate relationships. The beet chlorosis virus (BChV), a phloem‐restricted *Polerovirus* transmitted by *Myzus persicae* in a circulative and non‐propagative manner, represents an economically significant pathogen causing sugar beet yellowing and substantial yield reductions (Hossain et al. 2020). The re‐emergence of sugar beet yellowing disease, exacerbated by recent European restrictions on neonicotinoid insecticides use to control aphid populations, emphasises the need to better understand BChV‐mediated interactions between host plant and insect vector. In this study, we hypothesised that BChV infection may modulate plant metabolism and defence pathways in a way that could impact interactions with aphids and ultimately virus transmission. To address this, we combined targeted gene transcript analyses with metabolic profiling and aphid‐feeding behaviour monitoring, providing new insights into the molecular and genetic basis of plant–virus–vector relationships for an agronomically important pathosystem.

## 2 Materials and Methods

### 2.1 Virus, Aphids, and Plants

The beet chlorosis virus strain BChV‐2a was collected in England from sugar beet (Hauser et al. 2002). The virus was purified from infected spinach ( *Spinacia oleracea* ) following the procedure described in (van den Heuvel et al. 1991). The *Myzus persicae* (Sulzer) (Hemiptera: Aphididae) biotype WMp2 originated from the Netherlands (Reinink et al. 1989). Virus‐free aphids were reared on Chinese cabbage ( *Brassica rapa* *L. pekinensis var. Granaat*) in a growth chamber under 20°C ± 1°C and a 16 h photoperiod. The commercial variety of sugar beet named *Auckland* (SesVanderHave, Belgium) was kindly provided by the French Institute of Sugar Beet (Paris). Sugar beet plants were sown and grown in greenhouses until the first two true leaves developed (3 weeks after sowing). Subsequently, the plants were handled (e.g., for virus inoculation, aphid pre‐infestation, EPG experiments…) and grown in laboratories or growth chambers under 21°C ± 1°C and a 14 h photoperiod under LED lights.

### 2.2 Virus Inoculation, Aphid Pre‐Infestation, and Virus Detection

**FIGURE 1:**
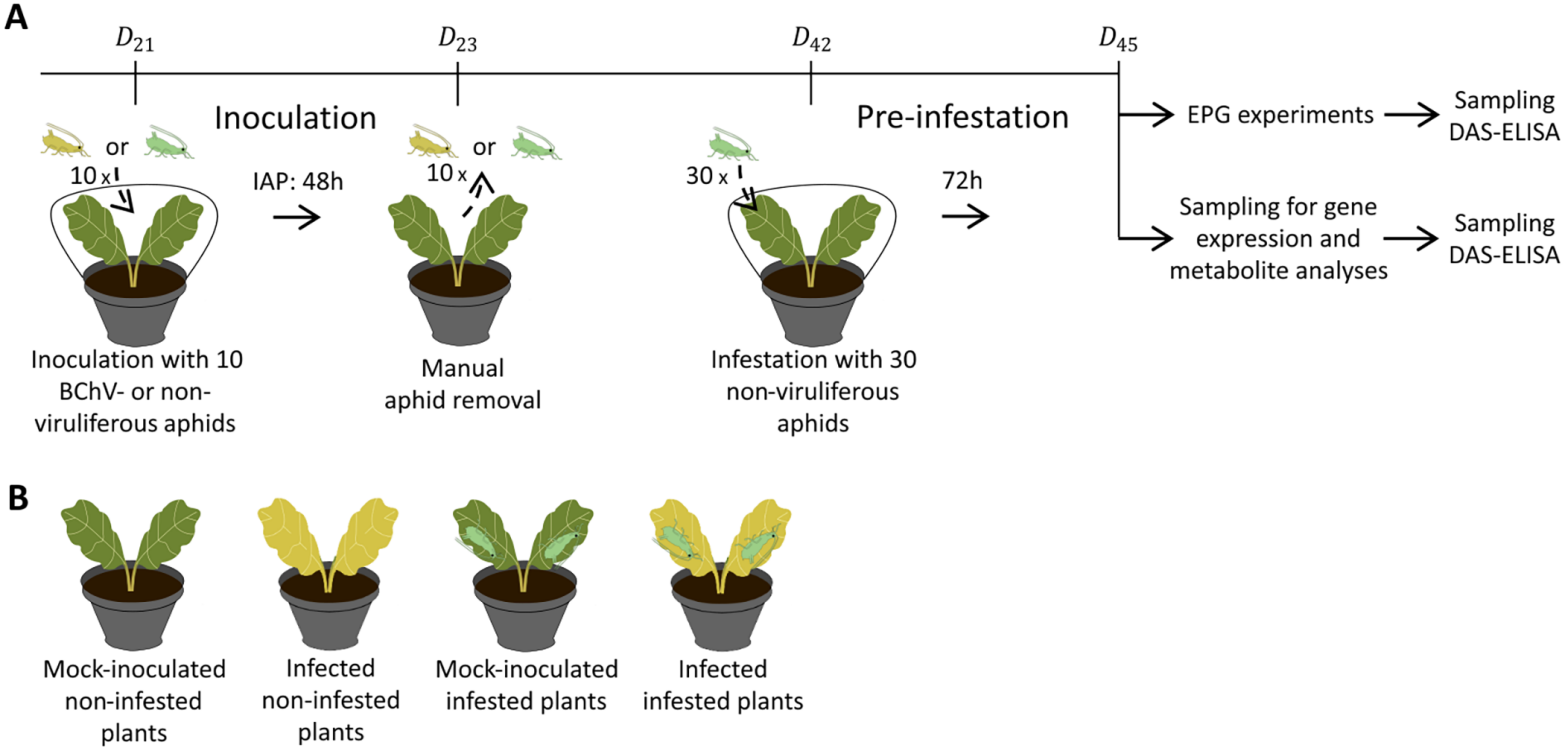
(A) The timetable for the experiment: At Day 0, the plants were sown and placed in the greenhouse. On Day 21 (*D_21_*), plants were inoculated with 10 BChV‐viruliferous aphids (or non‐viruliferous for ‘mock‐inoculation’) and placed in growth chambers. On Day 23 (*D_23_*), the aphids were manually removed. On Day 42 (*D_42_*), plants were pre‐infested with 30 non‐viruliferous aphids (or kept ‘non‐infested’). At Day 45 (*D_45_*), plants were used for EPG experiments or sampled for gene expression and metabolite analyses. EPG, Electrical Penetration Graph technique; IAP, Inoculation Access Period. (B) Four plant modalities used in experiments.

Virus‐free *M. persicae* were fed with an artificial medium (Harrewijn 1983). To obtain viruliferous aphids, purified BChV virions were added in artificial medium at a concentration of 50 ng/μL which is expected to result in an average of 50% of plants infected using the following conditions. After a 24 h acquisition access period, 10 aphids, either virus‐free or viruliferous, were transferred onto individual 3‐week‐old sugar beet plants (Day 21, Figure 1) for a 48‐h inoculation access period (Day 23). Aphids were then manually eliminated and the plants were maintained for 3 weeks in growth chambers. After 19 days (Day 42), mock‐ and BChV‐inoculated plants were either kept non‐infested or exposed to a pre‐infestation carried out with virus‐free *M. persicae* (i.e., 30 insects/plant for 72 h). At this time (Day 45), plants were used for EPG experiments, gene expression and metabolite analysis. The complete timetable and presentation of the four plant modalities are presented in Figure 1.

To validate the infected/non‐infected status of BChV‐ and mock‐inoculated plants respectively, the presence of BChV was assessed by DAS‐ELISA (Clark and Adams 1977). Because of high sequence similarities between the structural proteins of members in the *Polerovirus* genus (Hauser et al. 2002), a TuYV (referred to previously as beet western yellow virus, BWYV) polyclonal antiserum (LOEWE, Sauerlach; Germany) was used. Absorbance values were measured at 405 nm with a spectrophotometer Infinite F50 equipped with the software Magellan reader (Tecan, Männedorf, Switzerland). Samples were considered infected when the OD_405 nm_ is twice the mean value of the non‐infected control plant (i.e., 3 technical replicates) plus three times the standard deviation (Classen et al. 1987). BChV detection was carried out after the experiments so as not to influence the aphids' behavioural responses or molecular analyses carried out on Day 45.

### 2.3 Myzus persicae Feeding Behaviour Analysis on Sugar Beet Plants

The electrical penetration graph DC system (Tjallingii 1988) was used as described in (Chesnais et al. 2019). In brief, eight aphids were connected to the Giga‐8 DC‐EPG (electrical penetration graph technique) amplifier and each one was placed on the leaf of an individual sugar beet. The recordings were performed continuously for 8 h. Each aphid–plant system was placed inside a Faraday cage at 21°C ± 1°C. Acquisition and analysis of the EPG waveforms were carried out with PROBE 3.5 software (EPG Systems). Relevant aphid behaviour EPG parameters were calculated with EPG‐Calc 6.1 software (Giordanengo 2014). The following parameters were extracted from the EPG recordings based on their characteristic EPG waveforms described in (Tjallingii and Hogen Esch 1993): (Pr) stylet insertion and activity within plant tissues; (C) stylet pathways in plant tissues except for phloem and xylem; (E1) salivation in phloem elements; (E2) passive phloem sap ingestion; (G) active xylem sap ingestion. For each condition, 25 to 27 individuals were analysed, and aphids that had less than 5 h of activity (i.e., stylet insertion) were excluded from the analysis.

### 2.4 Metabolomic Analyses and Data Processing

Metabolite extraction was performed on ground freeze‐dried leaves (5 mg per sample) using 25 μL of methanol per mg of dry weight. The extract was then incubated in an ultrasound bath for 10 min before centrifugation at 13,000 g at 10°C for 10 min. The supernatant was analysed using a Dionex Ultimate 3000 UHPLC (Thermo Fisher Scientific, Waltham, USA) system. Chromatographic separations were performed on a Nucleodur C18 HTec column (150 mm × 2 mm, 1.8 μm particle size; Macherey‐Nagel, Germany) maintained at 30°C. The mobile phase consisted of acetonitrile/formic acid (0.1%, v/v, eluant A) and water/formic acid (0.1%, v/v, eluant B) at a flow rate of 0.3 mL.min^−1^. The gradient elution was programmed as follows: 0 to 1 min, 95% B; 1 to 2 min, 95% to 85% B; 2 to 7 min, 85% to 0% B; 7 to 9 min, 100% A. The sample volume injected was 1 μL. The UHPLC system was coupled to an Exactive Orbitrap (Thermo Fisher Scientific, Waltham, USA) mass spectrometer equipped with an electrospray ionisation (ESI) source operating in positive mode. Parameters were set at 300°C for ion transfer capillary temperature and 2500 V for needle voltage. Nebulisation with nitrogen sheath gas and auxiliary gas was maintained at 60 and 15 arbitrary units, respectively. Spectra were acquired within the m/z (mass‐to‐charge ratio) mass ranging from 100 to 1000 amu (a.m.u.) using a resolution of 50,000 at m/z 200 a.m.u. The system was calibrated internally using dibutyl‐phthalate as lock mass at m/z 279.1591, giving a mass accuracy lower than 1 ppm. The instruments were controlled using the Xcalibur software (Thermo Fisher Scientific, Waltham, USA). LC–MS‐grade methanol and acetonitrile were purchased from Roth Sochiel (Lauterbourg, France); water was provided by a Millipore water purification system. Chloramphenicol (Sigma‐Aldrich, Saint Quentin Fallavier, France) was used as an internal standard. Amino acids were identified by comparing their mass spectra and retention times to those of the corresponding commercial standards (Table S1). Flavonoids of beet leaves were identified based on Hegazi et al. (2020).

### 2.5 Quantitative RT‐PCR

Leaf samples (200 mg) were collected 24 days post‐inoculation and total RNA extraction was performed with a Nucleospin RNA plant kit (Macherey–Nagel, Dueren, Germany) according to manufacturer instructions. Total RNA purity and concentration were measured with Nanodrop ND 1000 spectrophotometer. Complementary DNA (cDNA) was synthesised with M‐MLV reverse transcriptase (Promega, Wisconsin, USA) starting with 2 μg of total RNA and oligodT (1 μL at 500 μg/mL). Forward and reverse primers (Table S2) were used to amplify the cDNA corresponding to the targeted genes (Table 1). Quantitative PCR reactions using *iTaq Universal SYBR Green Supermix* (Bio‐Rad, Hercules, CA, USA) were conducted on a CFX cycler (Bio‐rad, Hercules, CA, USA), initiated with a 3 min incubation at 95°C followed by 40 cycles of amplification (30 s at 95°C, 30 s at 60°C). Melting curve analysis was performed from 65°C to 95°C with 5 s of 0.5°C increments. Amplification specificity was assessed by sequencing PCR products and analysing the melting curves of PCR products. Among the eight candidate genes tested (Table S3), *SAND*, *TIP41* and *PP2A* were selected with three commonly used software (geNorm (Vandesompele et al. 2002), BestKeeper (Pfaffl et al. 2004), and Normfinder (Andersen et al. 2004)) and further used as reference genes (Table S2). PCR efficiencies (E) were determined (Table S4) for each primer pair with a standard curve plotted from five ten‐fold serial dilutions. Moreover, correlation coefficients (*R* ^2^) were calculated for each primer pair and turned out to be higher than 0.98, which fits the RT‐qPCR requirements (Svec et al. 2015). Five biological replicates per modality and 3 technical replicates per sample were used to analyse the relative expression (RE) of the gene of interest. RE was determined from Ct values, PCR efficiencies, following Hellemans et al. (2007), and an internal control loaded on each plate, according to the formula: $\mathrm{RE}=\frac{E_{\mathrm{gi}}^{\left({\mathrm{Ct}}_{\text{control}}-{\mathrm{Ct}}_{\mathrm{gi}}\right)}}{\sqrt[{f}]{\prod_{o}^{f}E_{{\mathrm{ref}}_{o}}^{\left({\mathrm{Ct}}_{\text{control}}-{\mathrm{Ct}}_{{\mathrm{ref}}_{o}}\right)}}}$.

**TABLE 1:** List of genes involved in biosynthesis and signalling of hormonal defence pathways.

Metabolic pathway	Gene names	Abbreviations	ID *A. thaliana* ^a^	ID *B. vulgaris* ^b^	Function	References
Jasmonic acid	*Allene oxide synthase*	*AOS*	At5g42650	104,906,974	Biosynthesis^c^	(Bera et al. 2020)
	*Jasmonoyl‐L‐amino acid synthetase*	*JAR1*	At2g46370	104,886,569	Signalling pathway^c^	(Arena et al. 2016)
	*Coronatine insensitive 1*	*COI1*	At2g39940	104,903,682	Signalling pathway	(Bera et al. 2020)
Salicylic acid	*Isochorismate synthase*	*ICS*	At1g18870	104,905,177	Biosynthesis^d^	(Patton et al. 2020)
	*Phenylalanine ammonia‐Lyase*	*PAL*	At2g37040	104,898,673	Biosynthesis	(Morkunas et al. 2011)
	*Non‐expressor of Pathogenesis‐related protein 1*	*NPR1*	At1g64280	104,909,044	Signalling pathway^d^	(Morkunas et al. 2011)
Ethylene	*ACC synthase*	*ACS*	At3g61510	104,886,974	Biosynthesis^e^	
	*Ethylene response sensor 1*	*ERS1*	At2g40940	104,908,984	Signalling pathway^e^	(Bak et al. 2019)
	*Ethylene insensitve 2*	*EIN2*	At5g03280	104,884,677	Signalling pathway	(Patton et al. 2020)
	*Phytoalexin deficient 1,2*	*PDF1.2*	At5g44420	104,897,590	Defense^e^	(Casteel et al. 2015)

^a^ GenBank ID of gene of interest in *Arabidopsis thaliana* .

^b^ GenBank ID of orthologous genes in *Beta vulgaris* (from KEGG).

^c^ From (Ruan et al. 2019).

^d^ From (Zhang and Li 2019) and (Klessig et al. 2018).

^e^ From (Binder 2020).

### 2.6 Statistical Analysis

Statistical analyses were carried out with R software (3.3.2 (http://www.r‐project.org/)) and the significance level of each test was fixed below 0.05. The effect of the tested factor(s) (here infection status, pre‐infestation status and their interaction) on measured variables (i.e., EPG parameters and RE of selected genes) was analysed using generalised linear models (GLM; package lme4; Bates et al. 2015) using a generic formula (formula = variable ~ infection status*pre‐infestation status). The fit of GLM was controlled by inspecting residuals and QQ plots. Relative gene expressions were normalised using Log2 transformation. Then, these data were used in Gaussian GLM (link = ’log’). Data on EPG ‘occurrence’ parameters (i.e., number of stylet insertions, number of xylem phases…) and ‘duration’ parameters (i.e., total duration of salivation in the phloem, total duration of phloem sap ingestion). Therefore, GLM adapted to the distribution of the data were used (‘occurrence’ parameters: GLM using a Poisson distribution (link = ’log’); ‘duration’ parameters: GLM using a Gamma distribution (link = ’inverse’)). Following GLM analysis, estimated marginal mean post hoc test (EMM; package emmeans; *p*‐value adjustment with Tukey method) was used to assess the significance of the difference between the studied groups. When no significant effect of ‘interactions’ was obtained in GLM analysis, post hoc test was carried out on the main factors (i.e., pre‐infestation and/or BChV infection) showing significant effect. Conversely, when ‘interaction’ was significant in GLM, post hoc test was performed on it.

For differential metabolomic analyses, Log2 fold changes were calculated using the means of peak areas in the samples of both experiments. Statistical analyses were performed using Tukey's honest significant difference method followed by a false discovery rate (FDR) correction using the Benjamini‐Hochberg procedure. Metabolites of interest were considered differentially accumulated when the FDR was below 5% (FDR < 0.05).

## 3 Results

### 3.1 The Combination of BChV Infection and Aphid Pre‐Infestation Alters Aphid Feeding Behaviour and Enhances Plant Acceptability

Using the EPG technique, we first looked at how BChV virus infection, aphid pre‐infestation, and the combined stresses affected the behavioural responses of the aphid vector, *Myzus persicae* . The four modalities tested here are summarised in Figure 1B. According to the ‘viral manipulation’ hypothesis, we hypothesised that viral infection may promote feeding behaviour responses in aphid vectors favourable to phloem‐restricted virus acquisition (i.e., prolonged stylet insertion and phloem sap ingestion), even in the context of plant infestation by aphids, which tends to reduce plant palatability for secondary aphid infestation (Escudero‐Martinez et al. 2021).

**FIGURE 2:**
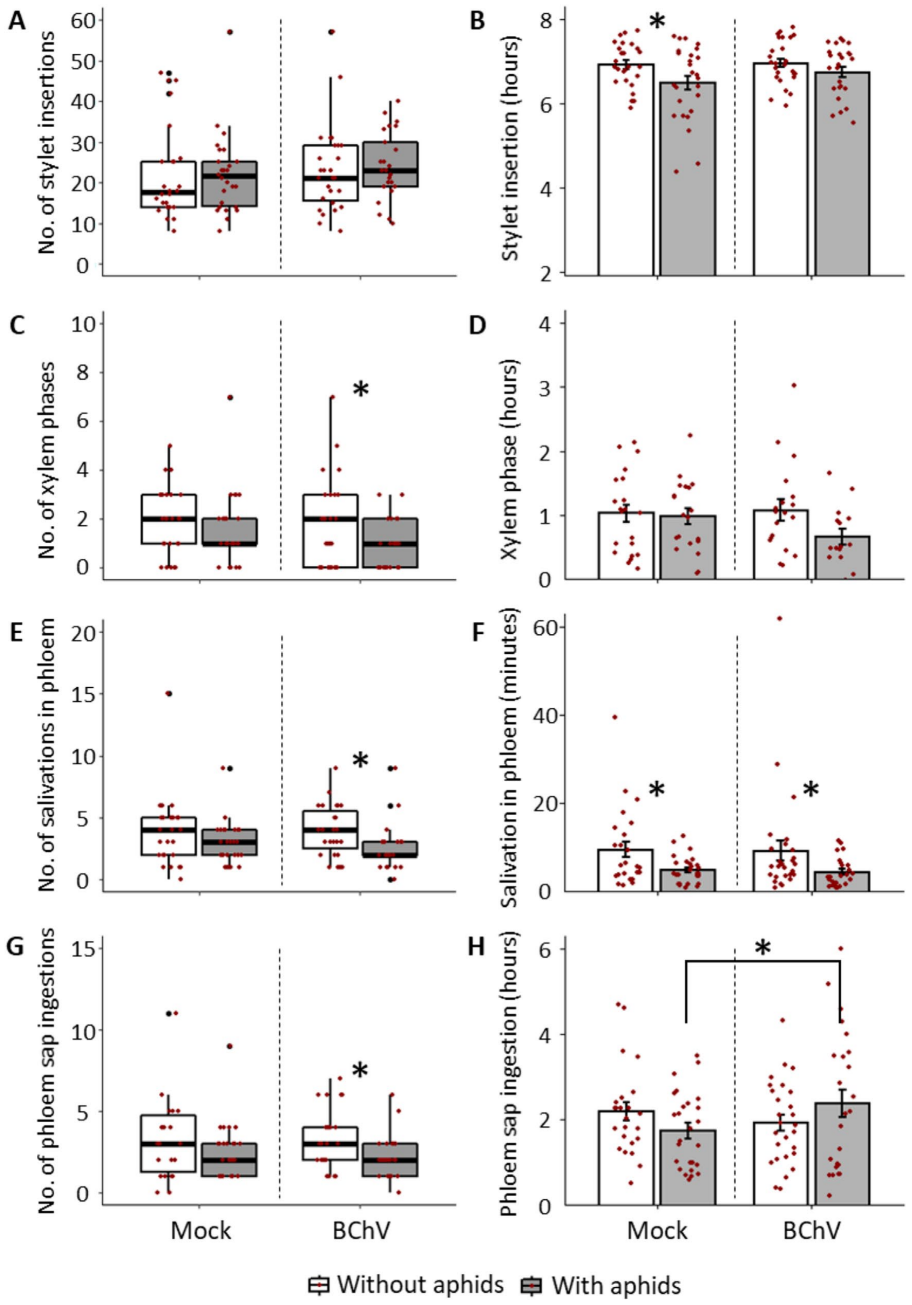
*Myzus persicae* feeding behaviour by EPG on sugar beet mock‐inoculated, BChV‐infected, pre‐infested, or not with aphids (*N* = 25–27). Asterisks indicate significant differences between conditions as tested by GLM (*p* < 0.05, Poisson (link = ’log’) distribution for ‘occurrence’ parameters and Gamma (link = ’inverse’) distribution for ‘duration’ parameters) (complete statistical analysis in Table S5). (A–H): Number (No.) or duration time of different phases of the aphid feeding behaviour.

In our experimental setup, aphids performed the same number of stylet insertions regardless of the infection and pre‐infestation status of sugar beets (Figure 2A, see Table S5 for complete statistical analysis). However, for mock‐inoculated plants, the total duration of the stylet insertion phase (probing time) in the plant was shorter for pre‐infested sugar beets compared to non‐pre‐infested ones (Figure 2B) as expected (Escudero‐Martinez et al. 2021). No such difference was observed when the sugar beets were infected with BChV (Figure 2B). The total duration of insertion of the stylets being associated with the plants' palatability for the aphid, this observation suggests that the BChV infection is counteracting the plant deterrence induced by an aphid pre‐infestation.

Poor plant palatability for aphids is often associated with behavioural phases in xylem and salivation in phloem (Spiller et al. 1990). Focusing on BChV‐infected plants, pre‐infestation reduced the number of xylem phases performed by aphids (Figure 2C). However, the total duration of the xylem ingestion phase was not altered by BChV infection nor aphid pre‐infestation (Figure 2D). The total duration of salivation in phloem tissues was reduced on pre‐infested plants, regardless of the infection status (Figure 2F). Similarly, aphids feeding on BChV‐infected plants performed fewer salivation phases in phloem when the sugar beets were pre‐infested (Figure 2E). Such a trend was not observed on mock‐inoculated sugar beets (Figure 2E). In agreement with data on stylet insertion, BChV‐infected sugar beets enhance plants' palatability for *M. persicae*. Surprisingly, for behavioural phases in xylem and salivation in phloem, the effect of BChV infection seems to be dependent on the plant's pre‐infestation by aphids.

For phloem‐restricted viruses, such as BChV, the ingestion of phloem sap by the vector is decisive for virus acquisition. Therefore, according to the ‘viral manipulation’ hypothesis, we expected that viral infection would maintain, or even favour, such behaviour. Pre‐infestation reduced the number of phloem sap ingestion phases by aphids on BChV‐infected plants but not on mock‐inoculated sugar beets (Figure 2G). BChV infection increased the total duration of phloem sap ingestions by aphids on pre‐infested plants compared to mock‐inoculated and infested sugar beets (Figure 2H). As expected by the viral manipulation hypothesis, such behaviour may promote the acquisition of BChV. Similarly, to behavioural phases in xylem and salivation in phloem, the effect of BChV infection seems to depend on the pre‐infestation status of the plants.

### 3.2 Aphid‐Induced Changes in Gene Expression Within the Ethylene Pathway Are Attenuated in BChV‐Infected Sugar Beets

The ethylene, jasmonate, and salicylate pathways are part of a conserved defence network in plants, making them key targets for investigating the hypothesis of viral manipulation (Mauck et al. 2019). Consequently, our study focused on these pathways to elucidate the molecular mechanisms underlying the observed changes in aphid feeding behaviour. More precisely, we monitored the relative expression (RE) of genes involved in the biosynthesis, the signalling and/or defence responses associated with these phytohormones.

**FIGURE 3:**
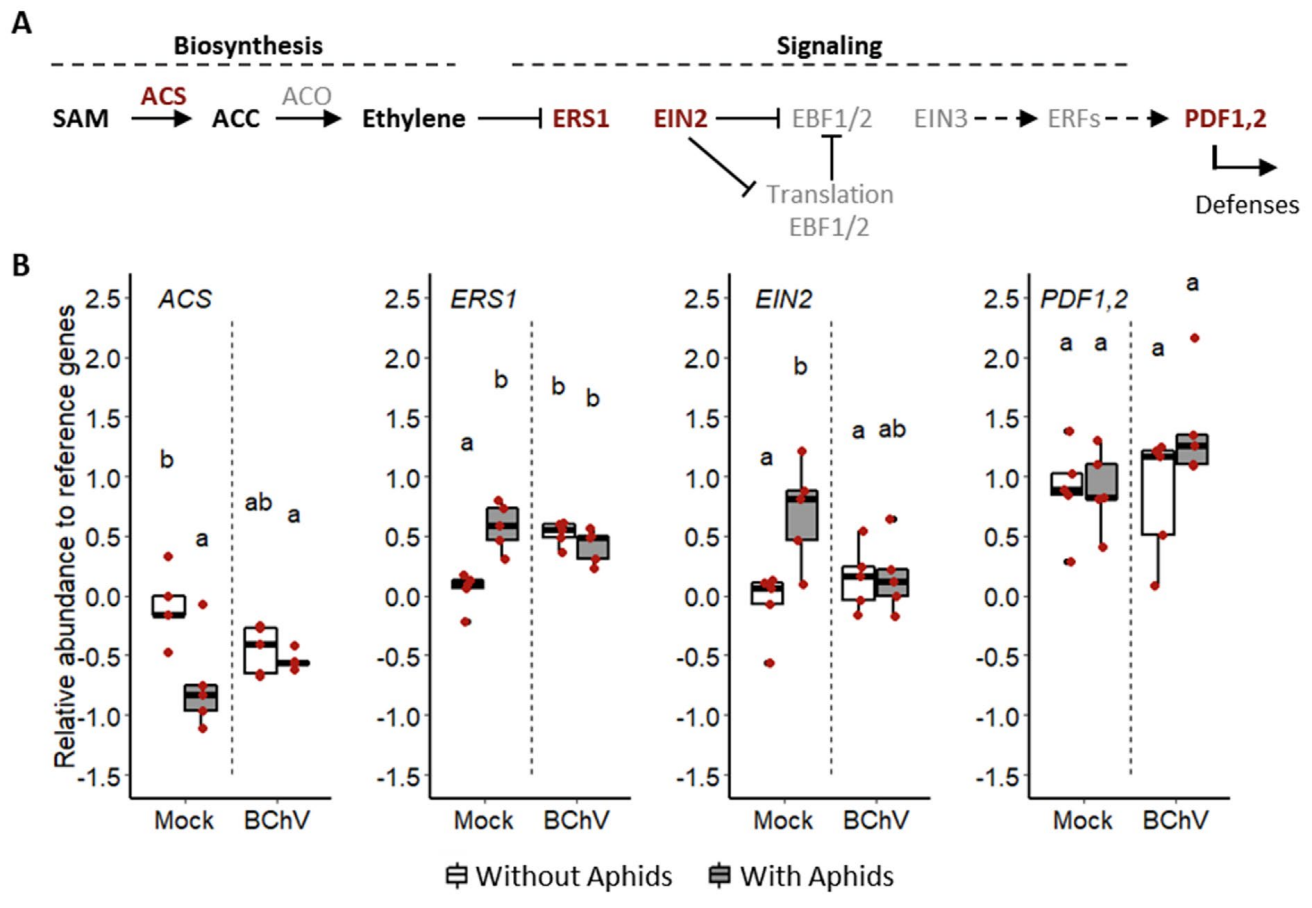
Simplified representation of the ethylene pathway adapted from (Binder 2020; Pattyn et al. 2021) (A); Relative gene expression (Log2) in mock‐inoculated, BChV‐infected sugar beets, pre‐infested or not with *Myzus persicae* (B). Each red dot corresponds to a plant sample (5 biological replicates for each condition). Box plots show median (black line), 25%–75% percentiles (box), and 10%–90% percentiles (whiskers). Different letters indicate significant differences between conditions as tested by GLM followed by pairwise comparisons using ‘emmeans’ (*p* < 0.05; method = ’Tukey’) (complete statistical analysis in Table S6). ACC, 1‐aminocyclopropane‐1‐carboxylic acid; ACO, ACC oxidase; ACS, ACC synthase; EBF1/2, Ethylene binding factor 1 or 2; EIN2, Ethylene insensitive 2; EIN3, Ethylene insensitive 3; ERFs, Ethylene response factors; ERS1, Ethylene response sensor 1; PDF1,2, Phytoalexin deficient 1,2; SAM, S‐adenosyl methionine.

**FIGURE 4:**
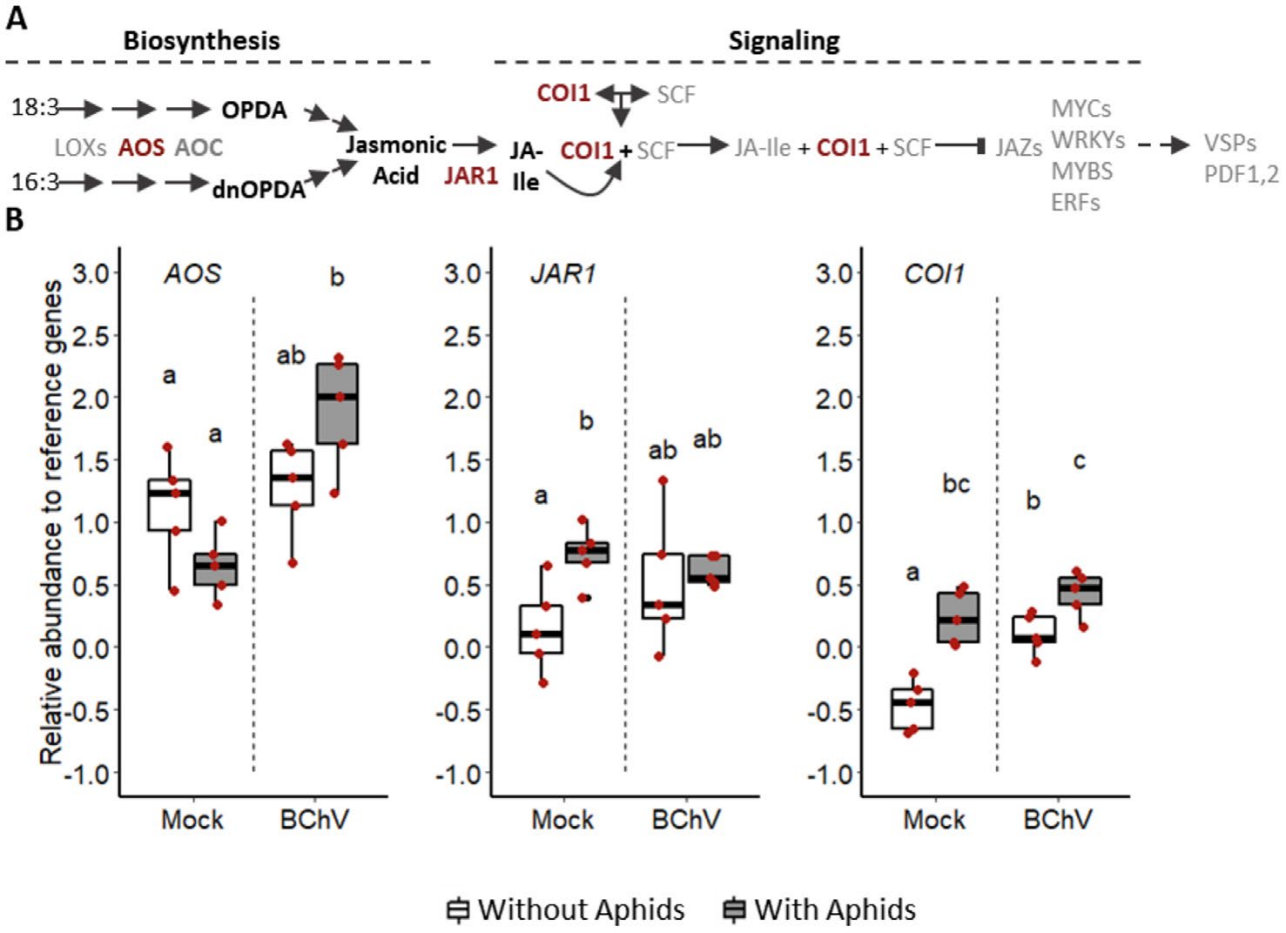
Simplified representation of the jasmonic acid pathway (Ruan et al. 2019) (A); Relative gene expression (Log2) in mock‐inoculated, BChV‐infected sugar beets, pre‐infested or not with *Myzus persicae* (B). Each red dot corresponds to a plant sample (5 biological replicates for each condition). Box plots show median (black line), 25%–75% percentiles (box) and 10%–90% percentiles (whiskers). Different letters indicate significant differences between conditions as tested by GLM followed by pairwise comparisons using ‘emmeans’ (*p* < 0.05; method = ’Tukey’) (complete statistical analysis in Table S7). 16:3, Hexadecatrienoic acid; 18:3, α‐linolenic acid; AOC, Allene oxide cyclase; AOS, Allene oxide synthase; COI1, Coronatine insensitive 1; dnOPDA, Dinor OPDA; ERFs, Ethylene response factors; JA‐ile, Jasmonoyl‐L‐isoleucine; JAR1, Jasmonoyl‐L‐amino acid synthase; JAZs, Jasmonate ZIM domain proteins; LOXs, Lipoxygenases; MYBs, MYB transcription factors; MYCs, MYC transcription factors; OPDA, 12‐oxo‐phytodienoic acid; PDF1,2, Phytoalexin deficient 1,2; SCF, Skp1, Culline and F‐Box proteins; VSPs, Vegetative storage proteins; WRKYs, WRKY transcription factors.

To assess the impact of BChV infection and/or *M. persicae* pre‐infestation on ethylene defence‐related pathway, the RE of four genes (*ACS*, *ERS1*, *EIN2*, and *PDF1*,*2*; Figure 4A) was monitored. These genes are involved in the ethylene pathway either in biosynthesis (*ACS*; Adams and Yang 1981), signalling (*ERS1* and *EIN2*; Alonso et al. 1999; Hall et al. 2000), or defence induction mediated by ethylene (*PDF1,2*; Penninckx et al. 1996) (Figure 3A). In our experimental conditions, we observed that the expression of the *PDF1,2* gene remained consistent across all modalities (Figure 3B). However, in mock‐inoculated sugar beets, the RE of *ACS* decreased following aphid pre‐infestation. In contrast, the RE levels of *ERS1* and *EIN2* were increased by pre‐infestation of mock‐inoculated plants. Interestingly, for ACS, ERS1, and EIN2, changes in RE associated with pre‐infestation in mock‐inoculated plants were not observed when the sugar beets were infected with BChV (Figure 3B).

### 3.3 BChV Infection Combined With Aphid Pre‐Infestation Increases the Expression of AOS in the JA Pathway

The RE of *AOS*, *JAR1*, and *COI1* genes involved in the jasmonic acid (JA) pathway (Gfeller et al. 2010; Katsir et al. 2008; Staswick et al. 2002) were monitored to evaluate the role of this pathway in *B. vulgaris* ‐BChV‐*M. persicae* interactions. *AOS* is involved in JA biosynthesis, while *JAR1* and *COI1* regulate JA signalling (Figure 4A). The combination of both BChV infection and pre‐infestation increased the *AOS* RE compared to the other tested modalities (Figure 4B). The RE of *JAR1* was elevated by aphid pre‐infestation in mock‐inoculated plants (Figure 4B). Such a pattern was not observed for BChV‐infected sugar beets. Lastly, *COI1* RE increased after aphid pre‐infestation, regardless of the infection status of the plants (Figure 4B).

### 3.4 Over‐Expression of PAL Gene Involved in SA Biosynthesis in BChV‐Infected Plants Is Alleviated by Aphid Infestation

**FIGURE 5:**
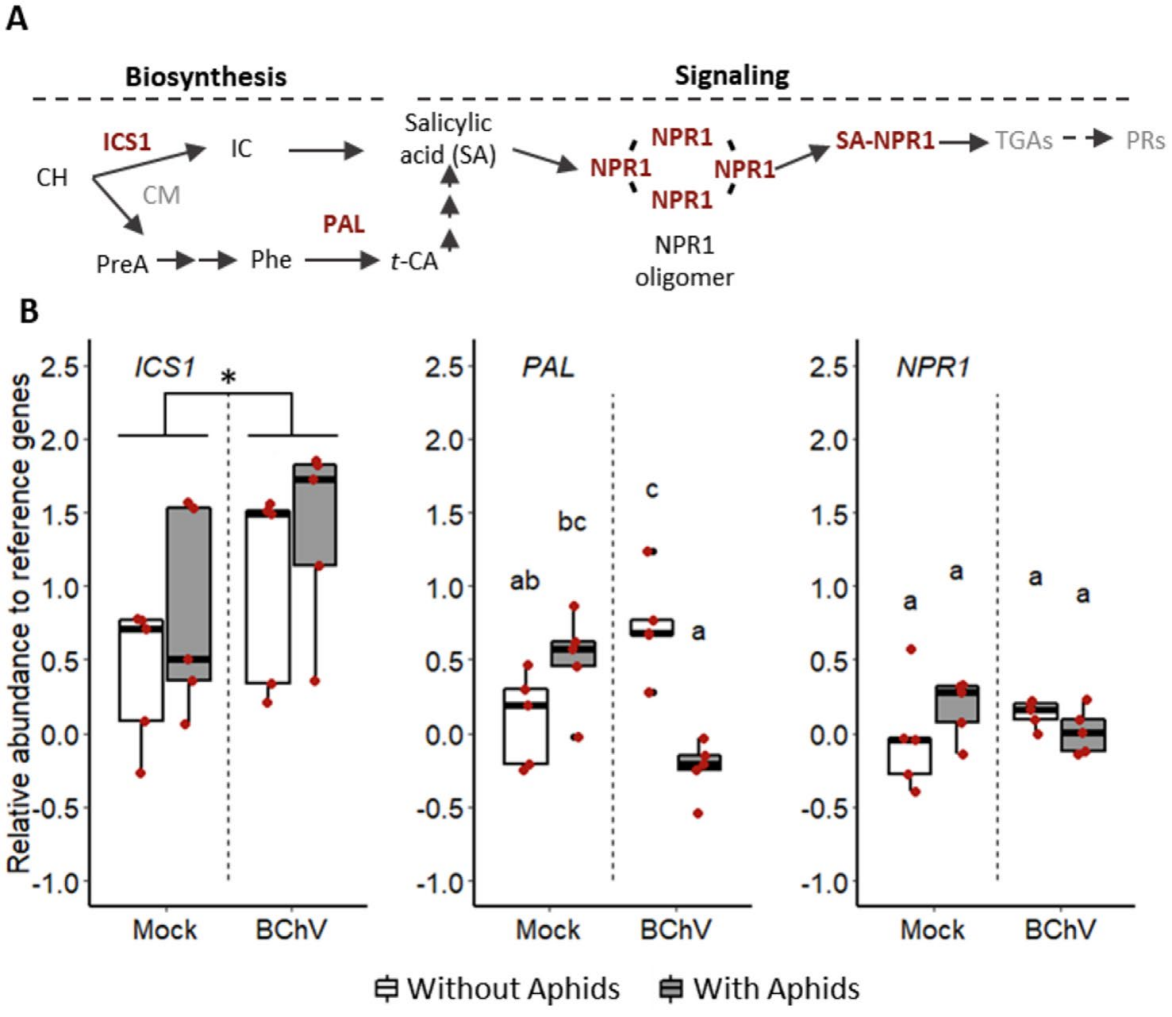
Simplified representation of the acid salicylic (SA) pathway (Klessig et al. 2018) (A); Relative gene expression (Log2) in mock‐inoculated, BChV‐infected sugar beets, pre‐infested or not with *Myzus persicae* (B). Each red dot corresponds to a plant sample (5 biological replicates for each condition). Box plots show median (black line), 25%–75% percentiles (box), and 10%–90% percentiles (whiskers). Different letters indicate significant differences between infection and infestation conditions as tested by GLM followed by pairwise comparisons using ‘emmeans’ (*p* < 0.05; method = ’Tukey’) (complete statistical analysis in Table S8). For ICS1, the asterisk indicates a significant difference between Mock and BChV‐infected plants (GLM, *p < 0.05; no significant interaction, infestation effect, or pairwise differences were detected). CH: Chorismate; CM: Chorismate mutase; IC: Isochorismate; ICS1: Isochorismate synthase; Phe: Phenylalanine; PAL: Phenylalanine ammonia‐Lyase; t‐CA: Trans‐cinnamic acid; NPR1: Non‐expressor of Pathogenesis‐related protein 1; PreA: Prephenic acid; TGAs: TGA transcription factors; PRs: Pathogenesis‐related proteins.

The RE of *ICS1*, *PAL*, and *NPR1* were analysed to investigate the role of the salicylic acid (SA) pathway in *B. vulgaris* ‐BChV‐*M. persicae* interactions. *ICS1* and *PAL* are involved in SA biosynthesis (Lefevere et al. 2020), while *NPR1* plays a key role in SA signalling (Wu et al. 2012) (Figure 5A). The RE of *ICS1* in sugar beets increased following BChV infection but was unaffected by aphid pre‐infestation (Figure 5B). The RE of *PAL* increased in sugar beets only infected with BChV. However, when BChV‐infected plants were pre‐infested by aphids, this increase of RE was mitigated (Figure 5B). Finally, *NPR1* expression was not influenced by either BChV infection or pre‐infestation by aphids (Figure 5B).

### 3.5 Metabolic Profiles Differentiated Pre‐Infested and Pre‐Infested/Infected Plants From Mock‐Inoculated Plants

In addition to monitoring changes in the expression of defence‐related phytohormone genes, metabolic analysis was carried out to deepen the exploration of the molecular pathway(s) involved in the observed changes in aphid feeding behaviour. These analyses were carried out on sugar beets exposed to conditions similar to EPG experiments (Figure 1B). Under such conditions, sugar beet plants infected with BChV and maintained in growth chambers exhibited mild symptoms 3 weeks post‐infection (Day 45), such as a slight reduction in above‐ground biomass combined with occasional mild yellowing on old leaves.

**FIGURE 6:**
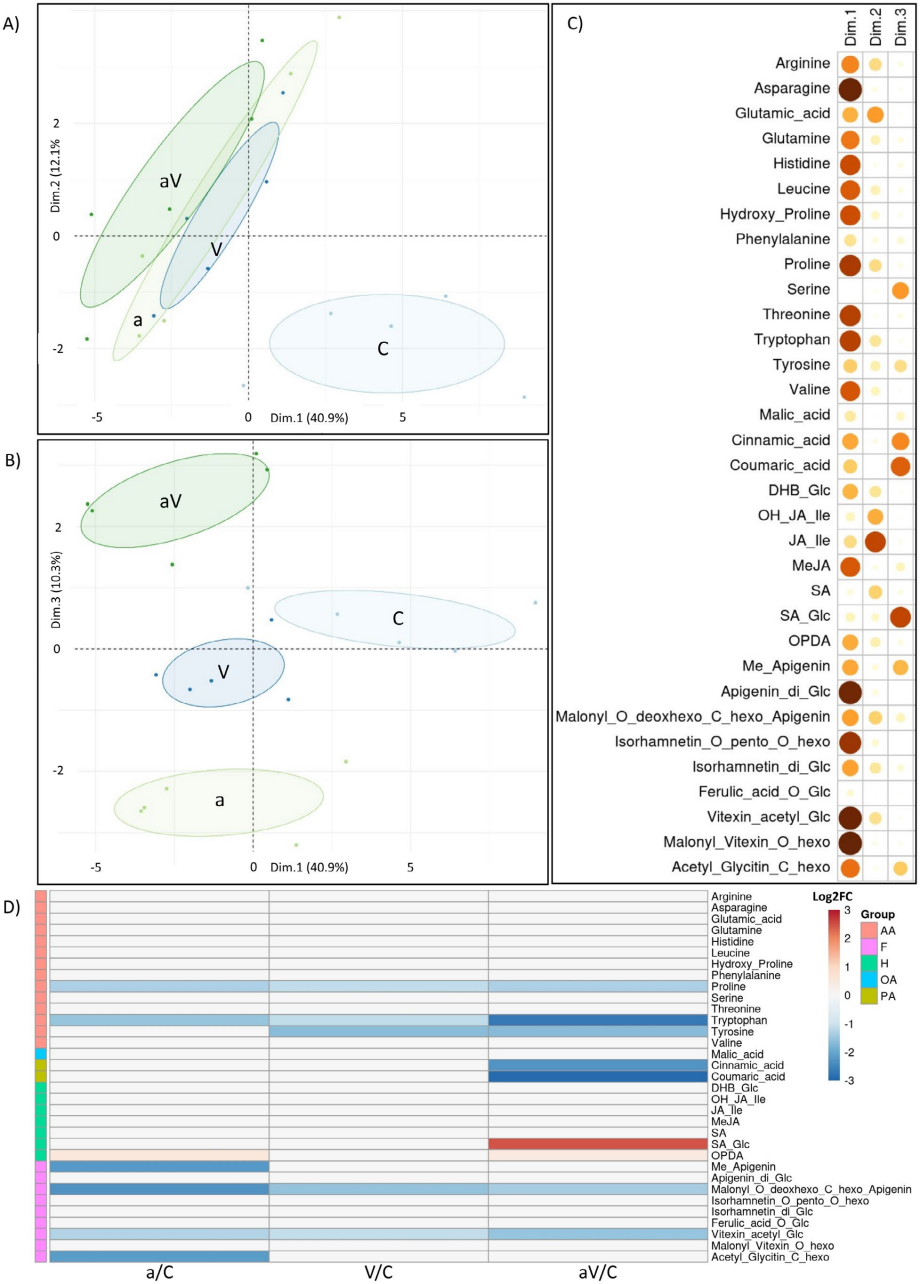
Principal component analysis (PCA) of metabolites in sugar beet following different treatments using the first two dimensions (A) or the first and the third dimensions (B). Contributions of the different metabolites in the three dimensions of the principal components (C). The contribution of each metabolite is relative to the circle size and the colour intensity, the largest and darkest ones contribute more. Heatmap of metabolite fold changes in sugar beet plants following the different treatments (D). Red and blue colours indicate respectively significant up‐ and down‐regulation in Log2 fold changes. Metabolites were grouped according to their chemical family as amino acids (AA), organic acids (OA), phenolic acids (PA), flavonoids (F), and hormones (H). Pairwise comparisons were performed using Tukey's Honest Significant Difference method followed by a false discovery rate (FDR) correction, with FDR < 0.05. For FDR ≥ 0.05, Log2 fold changes were set to 0. Non‐infected non‐infested control plants (C); BChV‐infected non‐infested plants (V); Mock‐inoculated aphid‐infested plants (a); BChV‐infected aphid pre‐infested plants (aV); Dim: Principal component. (*N* = 5).

Metabolic analysis focused on metabolites known, or suspected, to influence plant–aphid interactions (Mauck et al. 2016), including amino acids, hormones, organic acids, and flavonoids. Principal component analysis (PCA) was carried out to identify clusters of experimental modalities. The first two dimensions, representing 53% of the explained variance (Dim.1 = 40.9% and Dim.2 = 12.1%; Figure 6A), showed that the metabolic profile of control plants (C) formed a distinct cluster from stressed plants (i.e., BChV‐infected non‐infested plants (V), Mock‐inoculated aphid‐infested plants (a) or BChV‐infected aphid‐infested plants (aV)). Given that the second and third dimensions of this PCA explained comparable variability in the dataset (Dim.2 = 12.1% and Dim.3 = 10.3%; Figure 6A,B), a second PCA was conducted using the first and third dimensions (Figure 6B). This representation allowed for a distinct clustering of the metabolic profiles of the four modalities. Particularly, modality ‘a’ was differentiated from modality ‘aV’, indicating that the virus infection significantly influenced the metabolite composition of plants pre‐infested by aphids. Discrimination of different groups on the third axis was mainly driven by four metabolites: Salicylic acid glucoside (SA_Glc), Coumaric acid, Cinnamic acid, and Serine; and three additional contributors: Methylated Apigenin (Me_Apigenin), Acetylglycitin‐C‐hexoside (Acetyl_Glycitin_C_hexo) and Tyrosine (Figure 6C).

In addition to PCA, heat map analysis was performed to evaluate how BChV infection and/or aphid pre‐infestation affect the accumulation of metabolites in sugar beet leaves. To this aim, metabolite accumulation in stressed plants (i.e., modalities ‘a’, ‘V’ and ‘aV’) was compared to that of control mock‐inoculated plants (Modality ‘C’). This revealed a predominant decrease in metabolite accumulation compared to mock‐inoculated plants. This reduced accumulation was particularly pronounced for several amino acids such as proline and tryptophan in plants only pre‐infested by aphids, and proline, tryptophan and tyrosine in plants only infected by BChV (Figure 6D). These reduced accumulations were maintained when both stresses were combined (Figure 6D). For three other metabolites (Malonyl_O_deoxhexo_C_hexo_Apigenin, Vitexin_Acetyl_Glc and OPDA) changes in accumulation associated with infection and/or pre‐infestation were conserved when plants were exposed to both treatments (Figure 6D). However, this was not the case for two flavonoids (Me_Apigenin and Acetyl_Glycitin_C_hexo; Figure 6D), for which the combination of both pre‐infestation and BChV infection alleviated the decreased accumulation observed in plants of the modality ‘a’. Conversely, significant over‐accumulation of SA_Glc and decreased accumulation of two phenolic acids (i.e., cinnamic and coumaric acids) were only observed when plants were both pre‐infested by aphids and infected by BChV (Figure 6D). Altogether, these results showed that the combination of BChV infection and pre‐infestation by aphids induces specific changes in the metabolite profiles of sugar beets. Compared to plants only pre‐infested by aphids (‘a’), these changes include (i) lower accumulation of cinnamic acids, coumaric acids and tyrosine, (ii) accumulation of SA glycoside and (iii) basal accumulation of Methylated Apigenin and Acetyl Glycitin‐C‐hexoside.

## 4 Discussion

**FIGURE 7:**
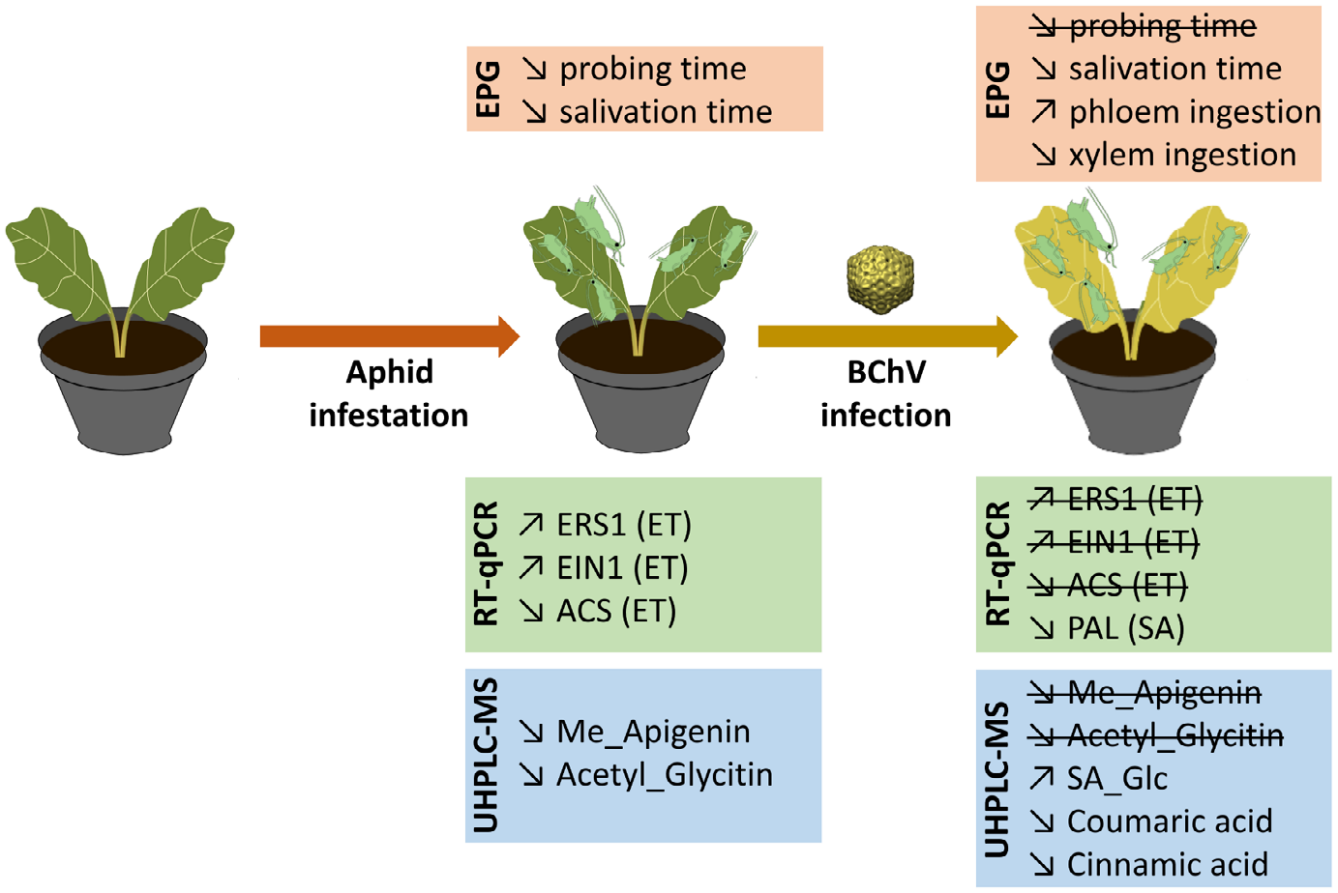
Summary of the effects of pre‐infestation and BChV‐infection, on aphid behavioural responses (EPG; orange), the expression of phytohormone‐related genes (RT‐qPCR; green) and accumulation of metabolites (UHPLC–MS; blue).

For most plant viruses, transmission is strongly influenced by insect behaviours, particularly the initial selection and acceptance of a host in a complex environment. This selection is mediated by plant‐emitted cues (Powell et al. 2006), the nature and quantity of which are altered in response to biotic and/or abiotic stresses (Fernando Gil et al. 2020; van Munster 2020; Verdier et al. 2025). Virus‐induced changes in plant cues can indirectly modify the behaviour and/or performance of insect vectors in ways that optimise plant virus transmission (‘virus manipulation’; Mauck et al. 2018). Despite an extensive literature on this subject, most of the data do not elucidate the molecular mechanisms underlying ‘virus manipulation’. Yet, deciphering such mechanisms is crucial for modelling virus spread in the field and for developing novel control strategies against plant virus diseases (Jeger 2020). In this context, our study focused on an agronomically relevant crop species (i.e., sugar beet) to investigate how BChV infection and aphid pre‐infestation affect the feeding behaviour of the vector, *M. persicae* (Figure 7). We also explored the potential involvement of phytohorme‐related defences as a molecular mechanism hijacked by BChV to facilitate its acquisition by aphids (Figure 7).

Modelling studies have shown that the prevalence of insect‐borne viruses in plant populations depends on the number of plants visited by viruliferous vectors (i.e., transmission frequency) and on the efficiency of each acquisition/inoculation event (i.e., transmission efficiency) (Cunniffe et al. 2021; Jones et al. 2010). These intrinsically linked parameters of transmission are influenced, in part, by various behavioural and biological traits of the vectors. Feeding behaviour analysis is therefore particularly well‐suited for investigating the ‘virus manipulation’ hypothesis as it encompasses distinct behavioural phases that are directly associated with (i) plant acceptability for aphids, and (ii) conditions required for efficient virus acquisition/inoculation.

According to the ‘virus manipulation’ hypothesis, circulative and phloem‐limited viruses, such as poleroviruses, are expected to increase plant acceptability for aphids, and facilitate phloem ingestion thereby increasing the efficiency of their own acquisition/inoculation (Mauck et al. 2018). Most available data on poleroviruses support this hypothesis (Chesnais et al. 2019; Mauck et al. 2018; Verdier et al. 2025). Our results are consistent with these findings but further suggest that such effects may depend on specific conditions, particularly on the simultaneous presence of both the virus and the vector on the plant. Indeed, on healthy plants, pre‐infestation with *M. persicae* altered several phases of aphid feeding behaviour in a way indicative of reduced plant palatability with frequent xylem feeding and reduced time in phloem phases (Spiller et al. 1990) (Figure 7). These observations align with previous studies conducted on *M. persicae* and other aphid species, on different host plants (Brunissen et al. 2009; Cardoza et al. 2005). However, this negative effect of pre‐infestation on plant palatability was alleviated on BChV‐infected plants, rendering BChV‐infected plants more comparable to uninfested healthy ones. Notably, the co‐occurrence of both virus and aphids on the same plant was associated with increased phloem ingestion time, an outcome favourable for virus acquisition (Brault et al. 2010) (Figure 7). In the same line, potato leafroll virus has been shown to accumulate at aphid feeding sites on infected leaves (Mowry 1995), suggesting that the virus might be able to ‘sense’ the presence of its vector and activate mechanisms to enhance its own transmission (a process referred to as ‘transmission activation’; (Drucker and Then 2015)). While this phenomenon is documented for several non‐circulative viruses (Berthelot et al. 2019; Martinière et al. 2013), evidence for such a mechanism for circulative viruses is limited. Our findings raise the possibility that a similar transmission activation process may occur in the sugar beet‐BChV‐ *M. persicae* interaction.

Exploring the molecular mechanisms underlying the ‘virus manipulation’ hypothesis, remains a long‐term objective that has been partially elucidated for a few pathosystems through the identification of key signalling pathways (Ziegler‐Graff 2020). Among these, phytohormone‐related defences (mostly JA, SA and ET‐pathways) have been extensively studied, as they are conserved across many plant species.

The JA‐pathway is primarily associated with plant defences against necrotrophic pathogens and herbivorous insects, including aphids (Moran and Thompson 2001). In our study, aphid pre‐infestation of healthy plants induced activation of the JA‐pathway (Figure 7), although no increased accumulation of JA‐derivative was observed under our experimental conditions. This pattern initially positioned the JA‐pathway as a potential mechanism through which BChV could counteract the negative impact of pre‐infestation. However, our results unexpectedly showed no suppression of JA‐mediated defences against aphids in BChV‐infected plants. These findings contrast with previous studies on other poleroviruses, in which virus infection mitigated the aphid‐induced activation of the JA‐pathway (Krieger et al. 2023; Patton et al. 2020; Pimenta et al. 2025). These discrepancies suggest that polerovirus effects on JA‐related defences may be host‐specific and time‐dependent, noting that our study examined only one time point.

SA is a key signalling molecule involved in plant defence responses against both biotic and abiotic stresses (Vlot et al. 2009). In our study, both *ICS1* and *PAL* transcripts, two genes associated with SA biosynthesis, were upregulated following BChV infection, but not after aphid pre‐infestation. This observation is consistent with previous studies showing that viruses can activate SA‐related pathways, sometimes to their own benefit (Murphy et al. 2020). Interestingly, in pre‐infested plants, BChV infection reduced *PAL* overexpression to basal levels. However, this transcriptional regulation did not translate into a reduction in SA or its derivative, SA‐glucoside. On the contrary, SA‐glucoside levels increased in plants subjected to both BChV infection and aphid pre‐infestation. Such SA‐glucoside is commonly observed during pathogen attacks, as SA glycosylation serves to store and detoxify free SA (Murphy et al. 2020). While this mechanism is generally protective for the plant (Dean et al. 2005), it can also be exploited by pathogens or herbivores to their advantage by lowering the concentration of free SA, an active component of plant defence. Thus, the observed accumulation of SA‐glucoside in BChV‐infected and aphid‐infested plants may reflect a synergistic interaction that enhances aphid feeding behaviour by reducing the toxic effects of free SA.

In addition to its involvement in the SA‐pathway, PAL is a key enzyme in the biosynthesis of phenylpropanoids, including flavonoids and phenolic acids (Vogt 2010). In our study, the relatively reduced expression of *PAL* in plants co‐exposed to BChV infection and aphid infestation was accompanied by a decrease in phenolic acids, specifically cinnamic and coumaric acids (Figure 7). This is noteworthy as derivatives of cinnamic and coumaric acids have been implicated in resistance or susceptibility to aphids in apple trees (Alonso‐Salces et al. 2022), and in response to aphid and beetle attacks in winter triticale (Czerniewicz et al. 2017). A coordinated effect of aphids and the virus was also observed on flavonoids accumulation, particularly for two compounds, Methylated Apigenin and Acetyl glycitin‐C‐hexoside. While levels of these flavonoids were reduced under individual stresses, their accumulation returned to basal levels in double‐treated plants, suggesting a compensatory response. Flavonoids are generally associated with anti‐insect properties (Kariyat et al. 2019; Lattanzio et al. 2000), although their effects can vary depending on the concentration (Simmonds 2003). For instance, while apigenin glycoside in alfalfa was shown to impair phloem sap ingestion and the performance of *A. pisum*, high concentrations of apigenin glycosides in artificial diets were shown to extend the phloem ingestion phase (Goławska et al. 2010). Thus, the observed changes could facilitate aphid feeding, potentially by extending the phloem sap ingestion phase and enhancing virus acquisition. These coordinated regulations in the phenylpropanoid metabolism suggest a possible joint manipulation strategy by the virus and the vector to suppress plant defences and optimise conditions for transmission.

Similarly to the JA‐pathway, the ET‐pathway has been shown to respond to aphid infestation (Mantelin et al. 2009; Wu et al. 2015), a pattern also observed in our study (Figure 7). Notably, in aphid‐pre‐infested plants, BChV‐infection appeared to mitigate changes in *EIN2* relative expression, a central regulator of the ET pathway (Figure 7). A similar modulation of *EIN2* expression has been reported in *N. benthamiana* and potato ( *Solanum tuberosum* ) plants exposed to either PLRV (*Polerovirus*), *M. persicae* or both (Bak et al. 2019). More recently, large‐scale transcriptomic analyses conducted in *A. thaliana* infected with TuYV (*Polerovirus*) showed that viral infection alleviated most of the plant defence against aphids, including ET‐related responses (Krieger et al. 2023). Beyond poleroviruses, similar strategies have been reported for non‐circulative viruses from the *Potyvirus* genus, which rely on the ET‐pathway to enhance attraction and/or fecundity of *M. persicae* on model (*A. thaliana* and *N. benthamiana*) or crop ( *Solanum tuberosum* ) species. Collectively, these findings suggest that the ET pathway represents a conserved regulatory node that aphid‐transmitted viruses may exploit to modulate vector behaviour and/or performance. Importantly, experimental evidence supports the practical relevance of this regulation. A single application of an ethylene biosynthesis inhibitor led to a 4‐fold reduction of PVY prevalence in multi‐host arenas (Bak et al. 2019). While further work is needed to validate this approach, targeting the ET pathway may represent a promising strategy for limiting the spread of aphid‐borne viruses in sugar beet.

## 5 Conclusion

Using sugar beet as an agronomically relevant model, we demonstrated that BChV infection can attenuate the plant defences against *M. persicae*, thereby enhancing plant acceptability for its aphid vector and potentially facilitating the virus's own acquisition. Indeed, BChV infection was associated with increased phloem sap ingestion, a critical step for the acquisition and transmission of this phloem‐limited virus. These behavioural changes in the feeding behaviour of the vector coincided with (i) increased accumulation of SA‐glycoside, (ii) altered levels of several phenylpropanoids and (iii) suppression of aphid‐induced responses in the ET‐pathway. Together, these findings point to a complex molecular framework through which BChV creates favourable conditions for its transmission by *M. persicae* . Although the specific defence pathways targeted by BChV differ in part from those affected by other viruses such as TuMV or PVY, our results support a broader and convergent evolutionary strategy among aphid‐transmitted viruses: manipulating plant defences and metabolism to adapt the aphid feeding behaviour to virus acquisition and, consequently, to virus spread. Finally, the use of sugar beet as a model crop offers valuable insights to identify molecular targets that could be leveraged through breeding or biotechnological strategies to reduce aphid‐mediated transmission of viruses in agricultural systems.

## Author Contributions

T.A., S.B., A.M.‐G., V.B., and Q.C. conceived the study; T.A. performed the feeding behavior analysis; T.A. and S.B. performed the molecular laboratory work; A.M.‐G. and P.H. performed the metabolomic analyses. T.A., S.B., A.M.‐G., P.H., V.B., and Q.C. analysed the data. T.A., S.B., A.M.‐G., V.B., and Q.C. wrote the initial draft. All authors revised and approved the manuscript.

## Conflicts of Interest

The authors declare no conflicts of interest.

## Supporting information


**Table S1:** List of the 33 metabolites detected in sugar beet leaves with ultra‐high‐performance liquid chromatography‐mass spectrometry (UHPLC–MS), using a targeted metabolomic approach.

**Table S2:** Primers used in RT‐qPCR experiments for amplification of target and reference genes.

**Table S3:** Candidate genes for reference genes.

**Table S4:** Amplification efficacy (%) of genes of interest and reference genes, and the associated correlation coefficient (R^2^).

**Table S5:** Detailed statistical analysis of *Myzus persicae* feeding behaviour by EPG on sugar beet mock‐inoculated, BChV‐infected, pre‐infested, or not with aphids.
**Table S6:** Detailed statistical analysis of the relative gene expression in the ethylene pathway.
**Table S7:** Detailed statistical analysis of the relative gene expression in the JA pathway.
**Table S8:** Detailed statistical analysis of the relative gene expression in the SA pathway.

## Data Availability

The authors confirm that all relevant data underlying the findings are fully available without restriction within the paper and its [Link], [Link], [Link], [Link], [Link]. Datasets on Quantitative RT‐PCR, metabolomic analyses and aphid feeding behaviour (EPG) have been made available in Recherche Data Gouv repository doi: https://doi.org/10.57745/L48YG6.
