# Supplementary material for: Beet Chlorosis Virus Infection Mitigates Aphid‐Induced Plant Defences and Improves Plant Acceptability to Aphid Vectors
Source: Mol Ecol. 2025 Aug 29;34(20):e70092. doi: 10.1111/mec.70092 (PMC12530290; doi:10.1111/mec.70092)
Supplement: Supplementary file 2 — Table S2: Primers used in RT‐qPCR experiments for amplification of target and reference genes. [file MEC-34-e70092-s003.docx]

**Table S2. Primers used in RT-qPCR experiments for amplification of target and reference genes**.

| **Gene names** | **Primer names** | **Sequence (5’→3’)** | **Tm (°C)** | **Size of PCR product (nt)** |
| --- | --- | --- | --- | --- |
| Defence gens |  |  |  |  |
| *ICS* | *F-ICS* | CCTCTCTTGCATACTGTGGCA | 64 | 90 |
|  | *R-ICS* | ATCGAATCCAAAGTGGACACA | 60 |  |
| *PAL* | *R-PAL1* | CTAAACAAGGTGGTGCCCTT | 60 | 94 |
|  | *F-PAL1* | GCAGTGTGTGGCTTGATTCT | 60 |  |
| *NPR1* | *F-NPR1* | GAAGCTTGTCGTCCTGCTGT | 62 | 98 |
|  | *R-NPR1* | AGGTGCCTCTGATAAAGCGA | 60 |  |
| *AOS*^a^ | *F-AOS* | ATTTGAACGGGCGGATGAGT | 60 | 92 |
|  | *R-AOS* | CCGGCCCATTAGACCAAACT | 62 |  |
| *JAR1* | *R-JAR1* | CTATTGGCTTGTCTGAGGTTGA | 64 | 91 |
|  | *F-JAR1* | GCATCTCCTAGCCTATAGCG | 62 |  |
| *COI1* | *F-COI1* | TGCACATTACTCGGTCTTGTTG | 64 | 95 |
|  | *R-COI1* | CGCCAGCTGATGAAGCCATT | 62 |  |
| ACS | *F-ACS* | GCGTTTAGAAATGCTGTGGC | 60 | 113 |
|  | *R-ACS* | CCATTAACTCATGGGCTCCA | 60 |  |
| *EIN2* | *F-EIN2* | AGTCGTCCAGAACTTTGGGG | 62 | 98 |
|  | *R-EIN2* | GGGGACATTGGAGTTCGAGG | 64 |  |
| *ESR1* | *F-ESR1* | CCCGTCTGATGGTGTAAGGA | 64 | 93 |
|  | *R-ESR1* | GCAGCATGAGAAAGAGCAACA | 60 |  |
| PDF1.2 | *F-PDF* | TCAAGACCAAGAAAAATGGAGAA | 60 | 95 |
|  | *R-PDF* | TCAACCTCAACCACCATGTT | 58 |  |
| Reference gens |  |  |  |  |
| *eEF1-α^b^* | *F-eEF1-α* | CGGCTAGGGTTCTTTCAGATTTGA | 66 | 103 |
|  | *R-eEF1-α* | TCGACTTTCCAGAGTCGACATG | 66 |  |
| *UKb* | *F-UK* | CGACACTCCGGTGCAAGTAAA | 64 | 109 |
|  | *R-UK* | CCACCGAGCACAAAAATGACG | 64 |  |
| *TIP41^b^* | *F-TIP41.1* | GCCAAAGTGAGAGTTATGCCCA | 66 | 93 |
|  | *R-TIP41.1* | ACGAGTGTCCCGTAGTCTCAT | 64 |  |
| *PUBQ^b^* | *F-PUBQ* | ACAGCCTATTCCCTTCATTGGT | 64 | 91 |
|  | *R-PUBQ* | AGATTTGCATCTTGAAAGAGAGGA | 66 |  |
| *SAND^b^* | F-SAND1 | CCCCTTGCAGACAAGGCTTTG | 66 | 110 |
|  | R-SAND1 | CACCAAGAGAAAGGGCTTGCT | 64 |  |
| *FBOX^b^* | *F-FBOX* | AACACAGAGAAGTCCGCAGC | 62 | 108 |
|  | *R-FBOX* | ATCGAGAAACTTGACTCAACGAGA | 66 |  |
| *PP2A^b^* | *F-PP2A* | AGTTGTTGGAGCCTCAGGAC | 62 |  |
|  | *R-PP2A* | GATTGGCAACCATGTACCGC | 62 | 99 |

a. No intron in AOS gene sequence.

b. For these genes, at least one of the two primers hybridizes on two introns. For the others, the primers hybridize on either side of an intron.
