# Supplementary material for: Beet Chlorosis Virus Infection Mitigates Aphid‐Induced Plant Defences and Improves Plant Acceptability to Aphid Vectors
Source: Mol Ecol. 2025 Aug 29;34(20):e70092. doi: 10.1111/mec.70092 (PMC12530290; doi:10.1111/mec.70092)
Supplement: Supplementary file 3 — Table S3: Candidate genes for reference genes. [file MEC-34-e70092-s005.docx]

| **Gene names** | **Abbreviations** | **ID *A. thaliana*^a^** | **ID *B. vulgaris*^b^** | **References** | **Used in** |
| --- | --- | --- | --- | --- | --- |
| Adenylate transporter | *ANT* | - | AF173648 | Chaouachi *et al.*, 2013 | GMO detection |
| Elongation factor 1-a | *eEF1-α* | At5g60390 | 104886831 | Liu *et al*., 2012 | Virus-plant interactions |
| F-box/kelch-repeat protein | *F-BOX* | At5g15710 | 104907176 | Liu *et al.*, 2012; Arena *et al.*, 2016 | Plant-virus-vector interactions |
| Protein phosphatase 2A | *PP2A* | At1g13320 | 104896530 | Liu *et al.*, 2012 | Virus-plant interactions |
| Polyubiquitine | *PUBQ* | DW505546 | 104907074 | Silva *et al.,* 2011 | *polerovirus*-Cotton interactions |
| Sand family protein | *SAND* | At2g28390 | 104906011 | Liu *et al.*, 2012; Arena *et al.*, 2016 | Plant-virus-vector interactions |
| TIP41-like family protein | *TIP41* | At4g34270 | 104904923 | Liu *et al.*, 2012; Arena *et al*., 2016 | Plant-virus-vector interactions |
| Uridylate kinase | *UK* | At5g26667 | 104906150 | Liu *et al.*, 2012 | Virus-plant interactions |

**Table S3. Candidate genes for reference genes**

1. GenBank ID. Alle ID come from *A. thaliana* genome except the DW505546 ID which comes from cotton.
2. GenBank ID of orthologous gens in *Beta vulgaris*.
