# Supplementary material for: Beet Chlorosis Virus Infection Mitigates Aphid‐Induced Plant Defences and Improves Plant Acceptability to Aphid Vectors
Source: Mol Ecol. 2025 Aug 29;34(20):e70092. doi: 10.1111/mec.70092 (PMC12530290; doi:10.1111/mec.70092)
Supplement: Supplementary file 4 — Table S4: Amplification efficacy (%) of genes of interest and reference genes, and the associated correlation coefficient (R2). [file MEC-34-e70092-s004.docx]

**Table S4. Amplification efficacy (%) of genes of interest and reference genes, and the associated correlation coefficient (R²).**

Efficacy expected values according litterature (De Keyser et al., 2013; Broeders et al., 2014; Veselenak et al., 2015; Shehata et al., 2019; Stephan, Tilmes, et Hülskamp, 2019) and according provider for R².

Abbreviations : ACS : Acid 1-aminocyclopropane-1-carboxylique synthase ; AOS : allene oxide synthase ; COI1 : Coronatine insensitive 1 ; EIN2 : Ethylene insensitve 2 ; ERS1 : Ethylene response sensor 1 ; ICS1 : Isochorismate synthase ; JAR1 : Jasmonoyl-L-amino acid synthetase ; NPR1 : Nonexpressor of Pathogenesis-related protein 1 ; PAL : Phénylalanine ammonia-Lyase ; PDF1,2 : Phytoalexin deficient 1,2 ; PP2A : Protein phosphatase 2A ; SAND : Sand family protein ; TIP41 : TIP41-like family protein and UK : Uridylate kinase.

| **Genes** | **Efficacy (%)** | **R²** |
| --- | --- | --- |
| **Expected values** | **80-120** | **≥ 0.980** |
| Reference genes |  |  |
| *PP2A* | 87,5 | 0,997 |
| *SAND* | 92,5 | 0,984 |
| *TIP41* | 96,4 | 0,983 |
| *UK* | 91 | 0,997 |
| Genes of interest |  |  |
| *AOS* | 97,3 | 0,986 |
| *ACS* | 81 | 0,981 |
| *COI1* | 87,1 | 0,998 |
| *EIN2* | 95,6 | 0,999 |
| *ERS1* | 95,2 | 0,992 |
| *ICS* | 96,5 | 0,992 |
| *JAR1* | 83 | 0.989 |
| *NPR1* | 91,7 | 0,998 |
| *PAL* | 80 | 0.991 |
| *PDF1,2* | 81 | 0,983 |
