# Supplementary material for: Beet Chlorosis Virus Infection Mitigates Aphid‐Induced Plant Defences and Improves Plant Acceptability to Aphid Vectors
Source: Mol Ecol. 2025 Aug 29;34(20):e70092. doi: 10.1111/mec.70092 (PMC12530290; doi:10.1111/mec.70092)
Supplement: Supplementary file 5 — Table S5: Detailed statistical analysis of Myzus persicae feeding behaviour by EPG on sugar beet mock‐inoculated, BChV‐infected, pre‐infested, or not with aphids. Table S6: Detailed statistical analysis of the relative gene expression in the ethylene pathway. Table S7: Detailed statistical analysis of the relative gene expression in the JA pathway. Table S8: Detailed statistical analysis of the relative gene expression in the SA pathway. [file MEC-34-e70092-s002.docx]

**Table S5.** Detailed statistical analysis of *Myzus persicae* feeding behavior by EPG on sugar beet mock-inoculated, BChV-infected, pre-infested, or not with aphids.

| **EPG parameters** | **Model** | **Infection status** | **Infestation status** | **Interaction infection:infestation** |
| --- | --- | --- | --- | --- |
| No. of stylet insertions | GLM(family = poisson) | *LR Chisq* = 3.404, *df* = 1, *P* = 0.065 | *LR Chisq* = 0.436, *df* = 1, *P* = 0.509 | *LR Chisq* = 0.121, *df* = 1, *P* = 0.728 |
| Stylets insertion (hours) | GLM (family = Gamma) | *LR Chisq* = 1.203, *df* = 1, *P* = 0.273 | *LR Chisq* = 6.376, *df* = 1, *P* = 0.012 * | *LR Chisq* = 0.791, *df* = 1, *P* = 0.374 |
| No. of xylem phases | GLM(family = poisson) | *LR Chisq* = 1.884, *df* = 1, *P* = 0.170 | *LR Chisq* = 6.325, *df* = 1, *P* = 0.012 * | *LR Chisq* = 1.197, *df* = 1, *P* = 0.274 |
| Xylem phases (hours) | GLM (family = Gamma) | *LR Chisq* = 0.683, *df* = 1, *P* = 0.409 | *LR Chisq* = 2.303, *df* = 1, *P* = 0.129 | *LR Chisq* = 2.360, *df* = 1, *P* = 0.124 |
| No. of salivations in phloem | GLM(family = poisson) | *LR Chisq* = 0.352, *df* = 1, *P* = 0.553 | *LR Chisq* = 9.503, *df* = 1, *P* = 0.002 ** | *LR Chisq* = 0.380, *df* = 1, *P* = 0.537 |
| Salivation in phloem (minutes) | GLM (family = Gamma) | *LR Chisq* = 0.078, *df* = 1, *P* = 0.780 | *LR Chisq* = 13.384, *df* = 1, *P* < 0.001 *** | *LR Chisq* = 0.089, *df* = 1, *P* = 0.765 |
| No. of phloem sap ingestions | GLM(family = poisson) | *LR Chisq* = 0.335, *df* = 1, *P* = 0.563 | *LR Chisq* = 6.082, *df* = 1, *P* = 0.014 * | *LR Chisq* = 0.443, *df* = 1, *P* = 0.506 |
| Phloem sap ingestion (hours) | GLM (family = Gamma) | *LR Chisq* = 0.685, *df* = 1, *P* = 0.408 | *LR Chisq* = 0.000, *df* = 1, *P* = 0.991 | *LR Chisq* = 4.173, *df* = 1, *P* = 0.041 * |

**Table S6.** Detailed statistical analysis of the relative gene expression in the ethylene pathway.

|  | **Model** | **Infection status** | **Infestation status** | **Interaction infection:infestation** |
| --- | --- | --- | --- | --- |
| *ACS* | GLM(family = gaussian) | *LR Chisq* = 0.384, *df* = 1, *P* = 0.536 | *LR Chisq* = 9.590, *df* = 1, *P* = 0.002 ** | *LR Chisq* = 5.225, *df* = 1, *P* = 0.022 * |
| *ERS1* | GLM (family = gaussian) | *LR Chisq* = 5.373, *df* = 1, *P* = 0.020 * | *LR Chisq* = 9.839, *df* = 1, *P* = 0.002 ** | *LR Chisq* = 21.588, *df* = 1, *P* < 0.001 *** |
| *EIN2* | GLM(family = gaussian) | *LR Chisq* = 1.156, *df* = 1, *P* = 0.282 | *LR Chisq* = 6.860, *df* = 1, *P* = 0.009 ** | *LR Chisq* = 6.495, *df* = 1, *P* = 0.011 * |
| *PDF1,2* | GLM (family = gaussian) | *LR Chisq* = 1.446, *df* = 1, *P* = 0.229 | *LR Chisq* = 2.051, *df* = 1, *P* = 0.152 | *LR Chisq* = 1.972, *df* = 1, *P* = 0.160 |

**Table S7.** Detailed statistical analysis of the relative gene expression in the JA pathway.

|  | **Model** | **Infection status** | **Infestation status** | **Interaction infection:infestation** |
| --- | --- | --- | --- | --- |
| *AOS* | GLM(family = gaussian) | *LR Chisq* = 15.936, *df* = 1, *P* < 0.001 *** | *LR Chisq* = 0.189, *df* = 1, *P* = 0.664 | *LR Chisq* = 9.511, *df* = 1, *P* = 0.002 ** |
| *JAR1* | GLM (family = gaussian) | *LR Chisq* = 0.507, *df* = 1, *P* = 0.476 | *LR Chisq* = 4.753, *df* = 1, *P* = 0.029 * | *LR Chisq* = 2.590, *df* = 1, *P* = 0.108 |
| *COI1* | GLM (family = gaussian) | *LR Chisq* = 19.977, *df* = 1, *P* < 0.001 *** | *LR Chisq* = 36.161, *df* = 1, *P* < 0.001 *** | *LR Chisq* = 4.990, *df* = 1, *P* = 0.026 * |

**Table S8.** Detailed statistical analysis of the relative gene expression in the SA pathway.

|  | **Model** | **Infection status** | **Infestation status** | **Interaction infection:infestation** |
| --- | --- | --- | --- | --- |
| *ICS1* | GLM(family = gaussian) | *LR Chisq* = 4.383, *df* = 1, *P* = 0.036 * | *LR Chisq* = 1.764, *df* = 1, *P* = 0.184 | *LR Chisq* = 0.004, *df* = 1, *P* = 0.952 |
| *PAL* | GLM (family = gaussian) | *LR Chisq* = 0.168, *df* = 1, *P* = 0.682 | *LR Chisq* = 4.423, *df* = 1, *P* = 0.035 * | *LR Chisq* = 26.057, *df* = 1, *P* < 0.001 *** |
| *NPR1* | GLM (family = gaussian) | *LR Chisq* = 0.001, *df* = 1, *P* = 0.970 | *LR Chisq* = 0.163, *df* = 1, *P* = 0.687 | *LR Chisq* = 2.533, *df* = 1, *P* = 0.112 |
